# Ketamine use in critically ill patients: a narrative review

**DOI:** 10.5935/0103-507X.20220027-en

**Published:** 2022

**Authors:** Thais Dias Midega, Renato Carneiro de Freitas Chaves, Carolina Ashihara, Roger Monteiro Alencar, Verônica Neves Fialho Queiroz, Giovana Roberta Zelezoglo, Luiz Carlos da Silva Vilanova, Guilherme Benfatti Olivato, Ricardo Luiz Cordioli, Bruno de Arruda Bravim, Thiago Domingos Corrêa

**Affiliations:** 1 Department of Critical Care Medicine, Hospital Israelita Albert Einstein - São Paulo (SP), Brazil.; 2 Department of Anesthesiology, Hospital Israelita Albert Einstein - São Paulo (SP), Brazil.; 3 Department of Critical Care Medicine, Hospital Municipal Dr. Moysés Deutsch - São Paulo (SP), Brazil.

**Keywords:** Ketamine, Anesthetics, Critical care, Deep sedation, Analgesia, Anesthesia

## Abstract

Ketamine is unique among anesthetics and analgesics. The drug is a rapid-acting general anesthetic that produces an anesthetic state characterized by profound analgesia, preserved pharyngeal-laryngeal reflexes, normal or slightly enhanced skeletal muscle tone, cardiovascular and respiratory stimulation, and occasionally a transient and minimal respiratory depression. Research has demonstrated the efficacy of its use on anesthesia, pain, palliative care, and intensive care. Recently, it has been used for postoperative and chronic pain, as an adjunct in psychotherapy, as a treatment for depression and posttraumatic stress disorder, as a procedural sedative, and as a treatment for respiratory and/or neurologic clinical conditions. Despite being a safe and widely used drug, many physicians, such as intensivists and those practicing in emergency care, are not aware of the current clinical applications of ketamine. The objective of this narrative literature review is to present the theoretical and practical aspects of clinical applications of ketamine in intensive care unit and emergency department settings.

## INTRODUCTION

Ketamine was described in 1965 and Food and Drug Administration (FDA) approved in 1970.^(1)^ The drug is an intravenous anesthetic with a variety of applications, including sedation, catalepsy, somatic analgesia, bronchodilation, and sympathetic nervous system stimulation.^(2)^

The use of ketamine in clinical practice was limited during a period of time due to its central nervous system side effects and characteristics of a drug of abuse.^(3)^ However, because of its hemodynamically stable profile, along with its beneficial respiratory properties and analgesic potency, it has recently made a resurgence.^(2)^ Thus, ketamine has been used in the treatment of postoperative and chronic pain, as a procedural sedative, and in the treatment of respiratory and/or neurologic clinical conditions, such as asthma and status epilepticus.^(4-7)^ The objective of this narrative literature review is to present the theoretical and practical aspects of the clinical application of ketamine, emphasizing its role in intensive care unit (ICU) and emergency department (ED) settings.

This was a narrative review of the literature about the clinical applications of ketamine. An electronic literature search was carried out in PubMed. The following search strategy incorporated keywords and utilized the following Medical Subject Headings: ((“ketamine”) and (“systematic” or “clinical trial” or “random allocation” or “therapeutic use”)). The present review included published studies from PubMed until January 2021.

The title and abstract from all the articles were scanned for relevance, and no restriction on language was adopted. The initial search strategy identified 5670 potentially relevant articles. Of those, 151 relevant articles were selected for a complete analysis. From these articles as well as from related reviews and meta-analyses, all references were inspected, and potentially relevant titles were hand searched.

## PHARMACOLOGY

### Pharmaceutics

Ketamine is a water-soluble phencyclidine derivative.^(8)^ The ketamine molecule contains an asymmetric carbon atom with two enantiomers: the S(+) isomer and the R(-) isomer.^(9)^ The S(+) enantiomer has more potent anesthetic/ analgesic activity, with a lower propensity for adverse reactions than the R(-) enantiomer. However, commercial preparations of ketamine are racemic mixtures.^(9)^ Ketamin can be administered orally, subcutaneously, intravenously (IV), intramuscularly, intranasally or intraosseously.^(4)^

### Mechanism of action and pharmacokinetics

Ketamine has several actions due to its versatility to interact with different body receptors.^(10)^ Ketamine is a rapid-acting general anesthetic that produces an anesthetic state characterized by profound analgesia, preserved pharyngeal-laryngeal reflexes, normal or slightly enhanced skeletal muscle tone, antidepressant effects, and occasionally transient and minimal respiratory depression.^(10)^ Its mechanism of action is mainly by noncompetitive antagonism of the N-methyl D-aspartic acid (NMDA) receptor.^(11)^ However, analgesia can also be mediated through serotonin and norepinephrine activation.^(12)^ It also interacts with opioid receptors, with a direct effect on delta opioid receptors and actions to augment opioid mu-receptor function.^(12)^

Ketamine stimulates the cardiovascular system, increasing heart rate, arterial blood pressure and cardiac output, mediated mainly through sympathetic nervous system activation, which promotes it as an attractive option to anesthetics with negative hemodynamic profiles.^(13)^ Ketamine also has an antagonistic interaction with monoaminergic, muscarinic, and nicotinic receptors, producing anticholinergic symptoms, such as bronchodilatation, salivation, and airway muscle tone increase.^(14)^ Ketamine induces cataleptic, amnestic, and profound analgesia and dose response anesthetic actions.^(10)^ The cataleptic state is an akinetic state with loss of orthostatic reflexes but without consciousness impairment.^(10)^

Ketamine easily crosses the blood-brain barrier, and it has an average onset of less than 5 minutes and average duration of 30 minutes.^(15)^ Ketamine is metabolized by the liver through N-demethylation via the cytochrome P450 system to form norketamine.^(8)^ This active metabolite is subsequently hydroxylated and excreted in the urine and feces as norketamine and hydroxylated derivatives.^(8)^

## ADVERSE EFFECTS

Ketamine is a safe and widely used drug, with few severe adverse effects reported.^(4)^ It is well established that all pharmacologic agents used for sedation can present some adverse effects.^(16)^ Among the pharmacologic agents routinely used for sedation, ketamine and propofol have been previously reported to have the lowest incidence of adverse events.^(16)^

Reported adverse effects with ketamine are commonly related to catecholamine release, increasing heart rate and systemic blood pressure, and to functional and electrophysiological dissociation between limbic systems and the thalamo-neocortical pathway, mainly dysphoria, hallucinations, disorientation, vivid dreams, sensory and/or perceptual illusions.^(2)^ Nevertheless, in adult patients, it has been demonstrated that sensory or perceptual illusions could be attenuated or prevented by the administration of benzodiazepine agents prior to ketamine infusion.^(17)^ Midazolam is the preferable benzodiazepine agent due to its shorter recovery time and minor adverse effects when prescribed in low doses and given as adjuvant therapy.^(18)^

Adverse effects such as sialorrhea and bronchorrhea may occur.^(14)^ Laryngospasm and apnea (with high doses or rapid administration) are rare adverse effects attributed to ketamine.^(14)^ They can be life-threatening and must therefore be monitored and rapidly treated.^(14)^ Due to the blockade of catecholamine reuptake, ketamine should be used with caution in patients with coronary artery disease and preexisting hypertension.^(4)^

## GENERAL CLINICAL USE

The most common uses of ketamine are facilitation of orotracheal intubation,^(19)^ management of acute and chronic pain,^(4)^ management of agitation and *delirium*,^(20)^ procedural sedation,^(5)^ refractory status epilepticus,^(21)^ ethanol abstinence,^(22)^ severe bronchospasm,^(6,14)^ traumatic brain injury and intracranial hypertension.^(23)^ Table 1 summarizes the most common clinical uses of ketamine.

**Table 1:** Most common uses of ketamine

	Considerations	Advantages/beneficial effects	Disadvantages/adverse effects	Proposed dose	Authors
Orotracheal intubation	Alternative for patients whose mental status led them to impede optimal preoxygenation and to manage anatomically difficult airways	1. Relative hemodynamic stability 2. Provides analgesia, amnesia, and sedation in a single agent 3. Allows continued spontaneous breathing	Risk of dissociative effects (hallucinations, disorientation, vivid dreams, sensory and/or perceptual illusions)	1.0mg/kg to 1.5mg/kg bolus IV	Merelman et al.^(19)^ Weingart et al.^(24)^ Jabre et al.^(25)^
Analgesia	Alternative for patients who no longer respond to high doses of opioids, patients with difficulty finding a suitable vein and perioperative analgesia	1. Reduces cumulative morphine consumption 2. Fewer adverse effects than opioids 3. Can be administered intramuscularly	Risk of dissociative effects (hallucinations, disorientation, vivid dreams, sensory and/or perceptual illusions)	0.25 to 0.5mg/kg bolus IV and 0.05 to 0.4mg/kg/h in continuous infusion	Cohen et al.^(4)^ Bell et al.^(26)^ Himmelseher et al.^(27)^ Lee et al.^(28)^
Agitation and *delirium*	Alternative to sedation in the prehospital setting, and a rescue medication in ED	1. Controls agitation faster than standard medications for delirium 2. Can be administered subcutaneously, and intramuscularly	May cause: 1. Hypersalivation, 2. Emergence reaction, 3. Laryngospasm, 4. Vomiting	3 to 5mg/kg bolus IM Mankowitz et al.(5) Hurth et al.(29) or 2mg/kg IV bolus	Mankowitz et al.^(5)^ Hurth et al.^(29)^
Procedural sedation	Alternative for elderly patients or in trauma, hypovolemia, and sepsis	1. Can be used in cases of hypovolemia, hypotension, and bronchospasm 2. Can be used in combination with propofol	May cause: 1. Agitation, 2. Vomiting, 3. Recovery reactions, such as confusion, anxiety and hallucinations	0.5 - 1mg/kg IV	Bellolio et al.^(30)^ Lemoel et al.^(31)^
Refractory status epilepticus	Alternative for patients with refractory epilepsy	1.Suitable for patients with hemodynamic instability 2. It does not increase ICP	1.Large prospective randomized trials are needed to test safety, efficacy, and dosing 2. The use of concurrent anesthetics with ketamine, often necessary to treat RSE, might lead to adverse effects, such as severe acidosis	2.0mg/kg I.V bolus and 1.5 - 5.0mg/kg/h in continuous infusion	Alkhachroum et al.^(7)^ Gaspard et al.^(21)^
Bronchospasm and asthma	Alternative in severe asthmaticus status refractory to conventional therapy	1. May reduce airway resistance, mean peak airway pressure, arterial partial pressure of carbon dioxide. 2. May increase partial pressure of oxygen and lung compliance.	1.There is no consensus about the optimum doses and duration of the infusion of ketamine infusion. 2. May increase airway secretions	0.1 - 2.0 mg/kg I.V bolus and 0.15 - 2.5mg/kg/h in continuous infusion	Esmailian et al.^(32)^ Goyal et al.^(14)^
Traumatic brain injury and intracranial hypertension	Does not increase intracranial pressure	1. May offer protection from cellular mechanisms of neuronal death 2. Relative hemodynamic stability	1. There is no evidence that ketamine is more efficacious than other sedatives. 2. Longer recovery after infusion was discontinued	0.8mg/kg/h in continuous infusion IV	Bourgoin et al.^(33)^ Roberts et al.^(34)^
Ethanol abstinence	Alternative for patients with severe withdrawal symptoms	Ketamine infusion is associated with: 1. Reduced use of GABA agonists, 2. Shorter ICU stay, 3. Fewer intubations	Risk of dissociative effects (hallucinations, disorientation, vivid dreams, sensory and/or perceptual illusions)	0.15 - 0.3mg/kg/h in continuous infusion until delirium resolved	Pizon et al.^(22)^ Wong et al.^(35)^

ED - emergency department; IM - intramuscular; IV - intravenous; GABA - gamma-aminobutyric acid; ICU - intensive care unit.

## OROTRACHEAL INTUBATION

Tracheal intubation during emergency airway management is usually performed in patients with respiratory insufficiency/ failure, an inability to protect the airway, and high metabolic demand.^(24)^ Rapid sequence intubation (RSI) involves the simultaneous administration of a sedative, an analgesic and a neuromuscular blocking agent, rendering the patient unconscious, pain relieved and paralyzed to ensure optimal conditions for endotracheal intubation.^(19)^ To prevent hypoxemia during the apneic period of RSI, it is critical to provide the patient with adequate preoxygenation.^(24)^ Patients who are uncooperative due to *delirium*, intoxication, or head trauma can be difficult to preoxygenate, as they might be noncompliant with the application of a face mask, attempts at delivering noninvasive positive pressure ventilation, or with other procedures.^(24)^

A technique to allow adequate preparation of delirious or combative patients for intubation could decrease the risk of hypoxemia and reduce peri-intubation morbidity and mortality.^(24)^ Delayed sequence intubation (DSI) could be performed in patients whose medical condition or mental status led them to impede optimal preoxygenation.^(36)^ Ketamine is an extremely adequate induction agent to perform DSI, since it allows continued spontaneous breathing and maintenance of airway reflexes.^(36)^ The recommended dosage of ketamine is 1mg/kg, followed by an additional dose of 0.5mg/kg, if necessary, until the patient exhibits signs of dissociation.^(36)^ The average intravenous dose of ketamine to facilitate preoxygenation during DSI is 1.4mg/kg.^(36)^ Most patients experienced significant improvement in oxygen saturation before intubation.^(36)^ No complications (apnea, emesis, cardiac arrest, or death) were observed.^(36)^ Ketamine-induced dissociation leads to the maintenance of airway reflexes and spontaneous breathing, in contrast to other sedatives, thus becoming the wiser choice for DSI.^(36)^

Ketamine-only breathing intubation is the use of dissociative-dose ketamine to facilitate endotracheal intubation in spontaneously breathing patients, with or without the addition of topical anesthesia.^(19)^ Ketamineonly breathing intubation allows endotracheal intubation to be performed while the patient continues breathing, and it is primarily useful in managing airways that are known or predicted to be anatomically difficult.^(19)^ These patients are typically managed in elective anesthesia settings using local anesthesia and fiberoptic bronchoscopy, but this technique requires time and patient cooperation as well as skills and equipment that may not be available to emergency or critical care settings.^(19)^

A study conducted in 2009 compared 469 patients requiring sedation for emergency intubation who received 0.3mg/kg of etomidate or 2mg/kg of ketamine for intubation.^(25)^ The percentage of patients with adrenal insufficiency was significantly higher in the etomidate group than in the ketamine group. All patients received IV succinylcholine (1mg/kg) immediately after the trial medication and continuous sedation with midazolam (0.1mg/kg/hour). No significant differences were noted between groups in maximum sequential organ failure assessment (SOFA) scores during the first 3 days in the ICU (the primary outcome), intubation conditions, various measures of catecholamine use, or 28-day mortality. Therefore, the authors concluded that “ketamine is a safe and valuable alternative to etomidate for intubation in critically ill patients, particularly in septic patients”.^(25)^

A more recent prospective randomized open-label study compared ketamine (1 - 2mg/kg) to etomidate (0.2 - 0.3mg/kg) for emergency endotracheal intubation and found that the primary outcome of Day 7 survival was greater in patients randomized to ketamine, whereas there was no significant difference in survival by Day 28.^(37)^

## ANALGESIA

Ketamine has been administered as a coanalgesic in palliative care patients in addition to opioids and coadjuvant drugs.^(26)^ Ketamine is now considered to be an essential adjuvant analgesic for refractory cancer pain, and it is on the World Health Organization’s essential drug list for patients who no longer respond to high doses of opioids or have predictable breakthrough pain.^(26)^

For analgesia, doses of ketamine are 0.25 to 0.5mg/kg bolus IV (may be repeated if necessary, at a maximum dose of 2mg/kg in a 30-minute period) and 0.05 to 0.4mg/kg/h in continuous infusion.^(27)^ Moreover, ketamine as an adjunct to morphine may improve the latter’s effectiveness in cancer pain.^(26)^ Ketamine may be effective in the treatment of chronic peripheral and central neuropathic pain, phantom and ischemic limb pain, fibromyalgia, chronic regional pain syndrome, visceral pain, and migraine.^(4)^

Management of perioperative analgesia can be complex and influenced by multiple factors. A recent review of randomized double-blinded clinical trials of IV ketamine added to opioids for postoperative pain analgesia found that a ketamine-opioid combination significantly reduced pain scores, cumulative morphine consumption, and postoperative desaturation in patients undergoing thoracic surgery.^(38)^

Lee et al. conducted a systematic review and metaanalysis to evaluate whether low-dose ketamine in the ED provides better analgesia with fewer adverse effects than opioids, and its results support the routine use of ketamine for the treatment of severe pain in the ED as a first treatment, since it was equivalent to morphine or fentanyl in all trials studied.^(28)^ Finally, the evidence is scarce regarding pain control with the use of ketamine in nonoperative patients in the ICU.^(4)^ Ketamine is a traditional and well-stabilized option for analgesia in burn dressing changes, during excision and grafting, and for sedation.^(39)^ Additionally, in patients with difficulty finding a suitable vein, as in the case of burn patients, ketamine may be used for intramuscular administration.^(4)^

## AGITATION AND *DELIRIUM*

Guidelines for the management of pain, agitation, and *delirium* in the ICU recommend the use of nonbenzodiazepine sedatives in critically ill patients.^(40)^ The latest guide on analgesia, sedation, and *delirium* does not routinely recommend the use of ketamine for the specific treatment of *delirium*.^(40)^ However, a recent review on the use of ketamine in critically ill patients suggests a possible role of such medication in the management of agitated patients, yet with low-grade evidence.^(29)^ Ketamine has some advantages of profound sedation that have been pointed out: it favorably preserves gastrointestinal motility and respiratory function, reduces the need for vasopressor therapy, including cases of head trauma, and reduces postoperative cognitive dysfunction.^(20)^ Complications can include hypersalivation, emergence reaction, laryngospasm, and vomiting.^(29)^ Recent studies have shown the efficacy of ketamine for sedation in the prehospital setting and as a rescue medication in the ED.^(5,41)^ A meta-analysis with 18 included studies representing 650 patients showed that a mean dose of ketamine of 315mg administered intramuscularly provides adequate sedation in the mean time of 7 minutes, while traditional antipsychotics and benzodiazepines have action surges within 15 to 30 minutes.^(5)^ Ketamine appears to be faster at controlling agitation than standard ED medications for *delirium* and agitation.^(5)^

A recent trial randomized patients to receive ketamine (2mg/kg/h) or placebo and analyzed the impact of ketamine infusion on opiate use in mechanically ventilated ICU patients.^(42)^ The addition of low doses of ketamine did not decrease the consumption of opiates but reduced the incidence and duration of *delirium* without affecting the mortality rate and length of stay.^(42)^ Additionally, small studies demonstrated that low doses of ketamine as an adjunctive strategy for surgical or trauma intubated patients can significantly reduce opioid and propofol use, respectively.^(40,43)^

## PROCEDURAL SEDATION

Recently, ketamine used individually or in combination with other sedatives has appeared as an option for sedation procedures in adult patients in EDs and ICUs.^(44)^ Ketamine successfully attenuates propofol-induced hypotension, so it may be advantageous for the elderly or in trauma in cases of hypovolemia or sepsis.^(44)^

In a systematic review and meta-analysis on different sedation strategies for procedures, the incidence of agitation and vomiting was higher with ketamine than with propofol, midazolam or etomidate. However, the incidence of vomiting was drastically reduced with the regimen of ketamine plus propofol.^(30)^ Apnea was more frequent with midazolam, and hypoxia was less frequent in patients receiving ketamine plus propofol compared to other combinations.^(30)^ Furthermore, a multicenter, randomized, double-blind study in which adult patients received ketamine or ketamine plus propofol as sedatives for emergency procedures found a significant reduction in the incidence of recovery reactions, such as confusion, anxiety or hallucinations, in the ketamine plus propofol group as well as emesis frequencies among adult patients.^(31)^

## REFRACTORY STATUS EPILEPTICUS

Refractory status epilepticus is defined as status epilepticus that does not respond to appropriate therapy with typical antiepileptic drugs, agonists of the gamma-aminobutyric acid (GABA) system, which have a neuronal inhibitory effect.^(7)^ After a prolonged convulsive state, GABA receptors are rapidly internalized, leading to a reduction in GABA-mediated synaptic inhibition.^(7)^ Thus, the potency of GABAergic agents reduces as the duration of the seizure increases, requiring higher doses that can produce serious adverse effects, especially hypotension.^(21)^

Based on the pathophysiology of the disease, ketamine has gained importance because it is a noncompetitive NMDA receptor antagonist.^(7)^ Studies have demonstrated its efficacy and safety for the treatment of refractory epilepsy, especially if there is hemodynamic instability, but they are based on case series, retrospective studies, and modeling. ^(7,21)^ Larger prospective randomized trials are needed to test the safety, efficacy, and dosing and to determine the potential use of ketamine, alone or in combination.^(7,21)^ Currently, the most accepted dose in the ketamine literature is an intravenous bolus of 2mg/kg, followed by a continuous IV infusion of 1.5 to 5mg/kg/h.^(21)^

## BRONCHOSPASM AND ASTHMA

Asthma exacerbation presents variable responses to therapy, and bronchospasm can range from spontaneous resolution until refractory status, requiring fast and invasive mechanical ventilation.^(45)^ Almost 4% of patients presenting with acute exacerbation of asthma who need to be hospitalized will require invasive mechanical ventilation.^(45)^

Ketamine has been empirically used in severe asthmaticus status refractory to conventional therapy.^(6,32)^ The role of ketamine in patients with bronchospasm or asthma exacerbation is promising but controversial.^(32)^ Ketamine was associated with the reduction of airway resistance and relaxation of airway smooth muscle, reduction of the mean peak airway pressure, reduction of partial pressure of carbon dioxide in arterial blood (PaCO_2_), increased partial pressure of oxygen in arterial blood (PaO_2_), and increased lung compliance in patients with asthma and bronchospasm.^(6)^

However, to date, there have been few prospective control trials validating the clinical use of ketamine in patients with severe bronchospasm and/or asthma exacerbation, and there is no consensus about the optimum dose and duration of ketamine infusion.^(6,32,46)^ The dose of ketamine in bolus usually ranges from 0.1mg/kg to 2mg/kg, and the continuous infusion ranges from 0.15mg/kg/h to 2.5mg/kg/h.^(14)^

## TRAUMATIC BRAIN INJURY AND INTRACRANIAL HYPERTENSION

Traumatic brain injury (TBI) represents a major cause of death and disability.^(47)^ The cornerstones of the management of patients with TBI consist of avoiding secondary brain injuries and allowing optimum conditions for natural brain recovery.^(48)^

Sedative drugs are frequently used to manage critically ill patients with TBI.^(33,34)^ Nevertheless, sedative agents should be used with parsimony, as they may cause adverse drug events, including hypotension, which can contribute to secondary brain injury.^(33,34)^ There has been no evidence thus far that one sedative agent is more efficacious than another to improve outcomes in patients with traumatic brain injury.^(34)^

As presented before, ketamine has been widely used in many clinical situations but is not frequently used in patients with brain injury.^(33)^ The main reason to avoid ketamine in brain injuries is the results obtained from a small and noncontrolled study suggesting that ketamine could increase intracranial pressure (ICP) as well as cerebral metabolic oxygen consumption.^(49)^ Nevertheless, these findings have not been confirmed in more recent studies.^(33,34,50)^ In patients with severe brain injury, ketamine in combination with midazolam was not associated with increased intracranial pressure or decreased cerebral perfusion pressure.^(33)^ Moreover, in another trial with patients with intracranial hypertension undergoing mechanical ventilation, ketamine successfully reduced ICP and avoided untoward ICP elevations during distressing interventions without lowering blood pressure and cerebral perfusion pressure.^(50)^ Finally, as the calcium conductance of the NMDA receptor could be a mediator for a deleterious cascade, ending in excitotoxicity from extracellular glutamate increase,^(8)^ ketamine, by antagonizing NMDA receptors and inhibiting glutamatergic transmission, may offer protection from cellular mechanisms of neuronal death.^(33)^

## ALCOHOL WITHDRAWAL

The treatment of ethanol abstinence is based on the administration of GABA antagonists such as barbiturates and benzodiazepines.^(22)^ Patients with mild-to-moderate withdrawal symptoms present good results with these agents; however, the subgroup of patients who develop severe withdrawal symptoms (i.e., *delirium tremens*) often require ICU-level care, large doses of GABA agonists, prolonged hospitalization, and mechanical ventilation.^(22)^ These severe cases are associated with high rates of hospital morbidity and costs, prolonged sedation and *delirium* related to the use of large doses of long-acting GABA agonists.^(22)^

Ketamine offers a potentially favorable pharmacological mechanism in patients with alcohol withdrawal syndrome because it does not result in prolonged sedation requiring mechanical ventilation or *delirium*, which are common effects with the use of benzodiazepines.^(22,35)^ A retrospective cohort study showed that ketamine infusion is associated with reduced use of GABA agonists (benzodiazepines and phenobarbital), shorter ICU stay, and fewer intubations.^(22)^

The recommended dose of IV ketamine is 0.15 - 0.3mg/kg/h in continuous infusion until *delirium* resolves. Based upon withdrawal severity and degree of agitation, a ketamine bolus (0.3mg/kg) can be provided prior to continuous infusion in some patients.^(22)^

## CONCLUSION

The primary mechanism of action of ketamine, N-methyl D-aspartic acid-mediated antagonism, is unique among anesthetics and analgesics. Ketamine is useful as an adjuvant in the multimodal management of acute perioperative pain to improve pain therapy, and it reduces postoperative requirements and side effects of opioids.

Ketamine may cease prolonged status epilepticus and has fast-acting antidepressant action; thus, several new indications are emerging. In emergency care, ketamine can be used as an important agent for orotracheal intubation; in neurology, it helps to control intracranial pressure; and in psychiatry, it is used in the management of agitation, *delirium*, and alcohol withdrawal. The use of ketamine is extending now beyond the field of anesthesia into pain, palliative care, intensive care, and procedural sedation. Therefore, it is of paramount importance that intensive care unit and emergency department physicians have knowledge about the mechanisms of action, pharmacokinetics, main clinical applications, and potential deleterious effects of ketamine.
